# Choice of first-line antiretroviral therapy regimen and treatment outcomes for HIV in a middle income compared to a high income country: a cohort study

**DOI:** 10.1186/s12879-016-1443-0

**Published:** 2016-03-03

**Authors:** Gordana Dragovic, Colette J. Smith, Djordje Jevtovic, Bozana Dimitrijevic, Jovana Kusic, Mike Youle, Margaret A. Johnson

**Affiliations:** Department of Pharmacology, Clinical Pharmacology and Toxicology, School of Medicine, University of Belgrade, Belgrade, Serbia; UCL Research Department of Infection and Population Health, Royal Free Campus, 1st Floor, Rowland Hill Street, London, NW3 2PF UK; Infectious and Tropical Diseases Hospital, School of Medicine, University of Belgrade, Belgrade, Serbia; Department of HIV Medicine, Royal Free London Hospital, London, UK

**Keywords:** HIV, Middle income setting, High income settings, First line antiretroviral therapy, Switching, Treatment outcomes

## Abstract

**Background:**

The range of combination antiretroviral therapy (cART) regimens available in many middle-income countries differs from those suggested in international HIV treatment guidelines. We compared first-line cART regimens, timing of initiation and treatment outcomes in a middle income setting (HIV Centre, Belgrade, Serbia - HCB) with a high-income country (Royal Free London Hospital, UK - RFH).

**Methods:**

All antiretroviral-naïve HIV-positive individuals from HCB and RFH starting cART between 2003 and 2012 were included. 12-month viral load and CD4 count responses were compared, considering the first available measurement 12-24 months post-cART. The percentage that had made an antiretroviral switch for any reason, or for toxicity and the percentage that had died by 36 months (the latest time at which sufficient numbers remained under follow-up) were investigated using standard survival methods.

**Results:**

361/597 (61 %) of individuals initiating cART at HCB had a prior AIDS diagnosis, compared to 337/1763 (19 %) at RFH. Median pre-ART CD4 counts were 177 and 238 cells/mm^3^ respectively (p < 0.0001). The most frequently prescribed antiretrovirals were zidovudine with lamivudine (149; 25 %) and efavirenz [329, 55 %] at HCB and emtricitabine with tenofovir (899; 51 %) and efavirenz [681, 39 %] at RFH. At HCB, a median of 2 CD4 count measurements in the first year of cART were taken, compared to 5 at RFH (p < 0.0001). Median (IQR) CD4 cell increase after 12 months was +211 (+86, +359) and +212 (+105, +318) respectively. 287 (48 %) individuals from HCB and 1452 (82 %) from RFH had an available viral load measurement, of which 271 (94 %) and 1280 (88 %) were <400 copies/mL (p < 0.0001). After 36 months, comparable percentages had made at least one antiretroviral switch (77 % HCB vs. 78 % RFH; *p* = 0.23). However, switches for toxicity/patient choice were more common at RFH. After 12 and 36 months of cART 3 % and 8 % of individuals died at HCB, versus 2 % and 4 % at RFH (p < 0.0001).

**Conclusion:**

In middle-income countries, cART is usually started at an advanced stage of HIV disease, resulting in higher mortality rates than in high income countries, supporting improved testing campaigns for early detection of HIV infection and early introduction of newer cART regimens.

## Background

Since the introduction of combination antiretroviral treatment (cART), morbidity and mortality in those living with human immunodeficiency virus (HIV) has dramatically decreased [1, 2]. Treatment has been particularly effective in high-income countries, where the full spectrum of antiretroviral drugs (ARVs) are widely available, along with early access to experimental drugs [2, 3]. However, HIV therapy options in low-middle and middle income countries remain limited. In contrast to high income settings, the choice of cART regimen predominantly depends on which antiretroviral drugs are available, regardless of those recommended in treatment guidelines [4, 5].

The Strategic Timing of Antiretroviral Treatment (START) study showed that immediate initiation of cART with a CD4+ T-cell count >500 cells/mm^3^ led to lower rates of serious AIDS-related and non-AIDS-related illnesses and death compared to deferring cART until the CD4 count reached 350 cells/mm^3^ [5]. Thus, timely HIV diagnosis and introduction of cART is of great importance. However, in reality HIV-testing rates in high income countries are higher compared to low, low-middle and middle income settings. Consequently, higher numbers of individuals with HIV are diagnosed late and start cART with more advanced disease in these settings, with resulting higher mortality rates [3–5].

The objective of this study was to compare the ARVs used in first line therapy, timing of cART initiation, frequency of monitoring, frequency of cARV switches and treatment outcomes between a middle income setting (Belgrade, Serbia) and a high income setting (London, UK).

## Methods

### Patients

This study included all previously antiretroviral-naive individuals with HIV initiating cART from January 1^st^ 2003 until 1^st^ June 2012. Participants from Serbia, a middle income country, were attendees at the HIV/AIDS Center of the University Hospital for Infectious and Tropical Diseases in Belgrade (HCB). This is the largest center in Serbia, caring for over 90 % of cART-treated individuals in the country. A retrospective notes review of suitable patients was performed for the purpose of the study. Patients from the United Kingdom, a high income country, were attendees at the Ian Charleson Day Centre, Royal Free London Hospital (RFH), whose patient population has a demographic profile that is broadly representative of the UK HIV epidemic. Details of this cohort are given elsewhere [6]. The Royal Free HIV Cohort has approval to analyse anonymous routinely collected data from the Royal Free Hospital and Medical School Research Ethics Committee via Chairman's action. Data collection at HCB was approved by the Clinical Centre of Serbia Ethics Committee, approval number 29/XI-5. At the time of analysis, follow-up was available until 1^st^ June 2012.

### Laboratory methods

CD4+ T-cells were quantified by flow cytometry in both centres. HIV-1 RNA plasma viral load (pVL) was measured by quantitative reverse transcriptase polymerase chain reaction (Ultrasensitive assay version 1.5, Roche Molecular Systems, Branchburg, NJ, USA), with a lower limit of detection of 50 copies/mL. At the HCB, pre-cART CD4 counts were available for most patients, whereas baseline pVL and genotypic resistance testing are not performed. At the RFH, pre-cART CD4 counts, pVL and resistance tests are routinely performed for all patients.

### ARV availability

In Serbia, the following ARVs were available during the study period: a) nucleoside reverse transcriptase inhibitors (NRTIs): zidovudine, lamivudine, didanosine, stavudine, abacavir, and the fixed dose combinations of zidovudine with lamivudine and abacavir with lamivudine; b) non-nucleoside reverse transcriptase inhibitors (NNRTIs): nevirapine and efavirenz; c) protease inhibitors (PIs): saquinavir, nelfinavir, indinavir, fosamprenavir, lopinavir/ritonavir, and low-dose ritonavir. Nelfinavir and indinavir were withdrawn in 2008. Enfuvirtide was registered in 2007 solely for highly treatment experienced individuals with previous treatment failure. Integrase inhibitors and CCR5 receptor antagonists were not available. Ritonavir-boosted darunavir was available as a compassionate treatment, and as such was used in a very few patients. During the study period there were no specific National HIV treatment guidelines available, but doctors followed the European AIDS Clinical Society (EACS) guidelines wherever possible.

All antiretrovirals available in Serbia were also available in the United Kingdom. In addition, the following antiretrovirals were available: a) NRTIs: tenofovir, emtricitabine, and the fixed dose combination tenofovir with emtricitabine; b) NNRTIs: etravirine; c) PIs: atazanavir, darunavir and indinavir; d) integrase inhibitors: raltegravir; e) fusion and entry inhibitors: enfuvirtide and maraviroc. In addition, other experimental drugs could be used as part of clinical trials. During the study period, clinicians followed current British HIV Association (BHIVA) guidelines [7].

### Statistics

Baseline was defined as the date of starting cART. We described the participant characteristics at baseline, focusing on the specific NRTI backbone and ‘third’ drug used in the initial regimen. Comparisons between HCB and RFH were made using chi-squared tests, Fisher’s exact tests and Mann-Whitney U tests, as appropriate. Loss-to-follow-up was defined as not engaging in care for a period of longer than 12 months, and standard Kaplan-Meier methods were used to estimate the percentage to follow-up after 12 months.

The frequency of viral load and CD4 count monitoring in the first 12 months of ART were compared between the two settings. Immunological and virological response to cART was calculated after 12 months of ART, by considering the first CD4 count and pVL measurement recorded in the time period 12 to 24 months after start of ART. This definition was chosen as it is an analysis approach that is relatively insensitive to differences in frequency of monitoring between groups. A pVL cut-off of 400 copies/ml was considered so that low-level ‘blips’ were not considered as lack of virological response.

Clinician-defined reasons for switch were categorized into the following groups: virological failure, toxicity, dose reduction and/or ART simplification, patient choice, other reasons and not recorded. Switches for patient choice were only made at RFH. An additional reason for switch, only applicable in Serbia, was lack of drug supply, assigned when a particular antiretroviral was unavailable and so patients were prescribed an alternative (i.e. available) drug(s). Here, a drug from the combination would be replaced with a similar one (e.g. tenofovir with zidovudine or didanosine; lopinavir/r with fos-amprenavir; efavirenz with nevirapine). Switches were always made in accordance with antiretroviral history, previous toxicity, and results of previous genotypic HIV resistance tests if available.

Time to first switch of any drug in the cART regimen for any reason and for toxicity reasons were investigated using Kaplan-Meier methods, and the percentage that had made a switchby 36 months (a time point at which sufficient numbers remained under follow-up to allow accurate estimation) was reported. Individuals were followed from baseline until the date of the first change made to the cART regimen for any reason (or until the date of a switch made for toxicity reasons, ignoring any prior switches made for other reasons), or date of the last available pVL measurement, whichever occurred first. Two additional analyses were performed. Firstly, a time to first switch for any reason was re-performed for those from HCB, excluding “lack of drug supply” as a reason for stopping. Secondly, although “patient choice” was included as a ‘toxicity’ in primary analysis (as this reason represents tailoring regimens to an individual’s specific needs), a secondary analysis excluding “patient choice” as a “toxicity” discontinuation was also performed. Time to death was similarly investigated using Kaplan-Meier methods. All p-values of less than 0.05 were considered to demonstrate statistical significance. Analyses were performed using SAS Version 9.3 (SAS Institute Inc, Cary, NC).

## Results

### Baseline characteristics

There were 597 individuals from the HCB and 1763 individuals from the RFH who started cART during the study period, with a median (IQR) follow-up of 1.3 (0.4, 2.3) years and 3.4 (1.4, 5.6) years respectively. At the time of treatment initiation, those from the HCB had more advanced disease, with 361 (61 %) having a prior AIDS diagnosis (28 tuberculosis, 26 oesophagael candidiasis, 25 wasting syndrome) compared to 337 (19 %; 110 tuberculosis, 64 PCP, 33 Kaposi’s Sarcoma, 26 oesophageal candidiasis) at the RFH (Table 1). In addition, the median (IQR) pre-cART CD4+ T-cell counts was 177 (85, 298) cells/mm^3^ at HCB compared to 238 (123, 339) cells/mm^3^ at RFH.

**Table 1:** Patients characteristics at commencement of antiretroviral therapy in the HIV Centre Belgrade (HCB) and Royal Free Hospital (RFH) London

		HCB	RFH	P-value
Number		597 (100 %)	1763 (100 %)	
Gender	Male	478 (80 %)	1234 (70 %)	<0.0001
	Female	119 (20 %)	529 (30 %)	
Age	Median (IQR)	38 (32-44)	36 (32-43)	0.05
Ethnicity	White	597 (100 %)	892 (51 %)	<0.0001
	Black African	0 (0 %)	530 (30 %)	
	Other	0 (0 %)	341 (19 %)	
Risk for HIV Acquisition	Sex between men	218 (37 %)	850 (48 %)	<0.0001
	Heterosexual	155 (26 %)	839 (48 %)	
	Injecting Drug Use	90 (15 %)	49 (3 %)	
	Other	134 (22 %)	25 (1 %)	
Calendar year	2003-2005	268 (45 %)	682 (39 %)	
	2006-2008	126 (21 %)	648 (37 %)	
	2009-2012	203 (34 %)	433 (25 %)	
Previous AIDS	Yes	361 (61 %)	337 (19 %)	<0.0001
CD4+ T-cells count (cells/mm^3^)	Median (IQR)	177 (85, 298)	238 (123, 339)	<0.0001
		(N = 575)*	(N = 1519)**	
pVL (log_10_ copies/mL)	Median (IQR)	-^+^	4.9 (4.3, 5.4) (N = 1466)***	-
Previous TB diagnosis	Yes	28 (5 %)	127 (7 %)	0.03
HCV Ab status	Positive	131 (22 %)	88 (5 %)	<0.0001
	Negative	466 (78 %)	1005 (57 %)	
	Unknown	0 (0 %)	688 (39 %)	
HBV sAg status	Positive	78 (13 %)	53 (3 %)	<0.0001
	Negative	56 (87 %)	1005 (57 %)	
	Unknown	0 (0 %)	705 (40 %)	

IQR = inter quartile range; TB = tuberculosis; pVL = plasma HIV RNA viral load; HCV Ab = Hepatitis C virus antibody; HBV sAg = hepatitis B virus surface antigen; *available for 575 patients, **available for 1519 patients ***available for 1466 patients; ^+^ pre-cART pVL measurements not performed at HCB

Over calendar time, increases in the median pre-cART CD4+ T-cell counts were observed at both centres. At HCB, the median pre-ART CD4+ T-cell count was 110 cells/mm^3^ amongst those starting cART in 2003, increasing to a median of 197 cells/mm^3^ in 2007 and 298 cells/mm^3^ in 2012. Equivalent figures for RFH were 208 cells/mm^3^ in 2003, 225 cells/mm^3^ in 2007 and 319 cells/mm^3^ in 2012.

### Components of cART regimen

**Fig. 1:**
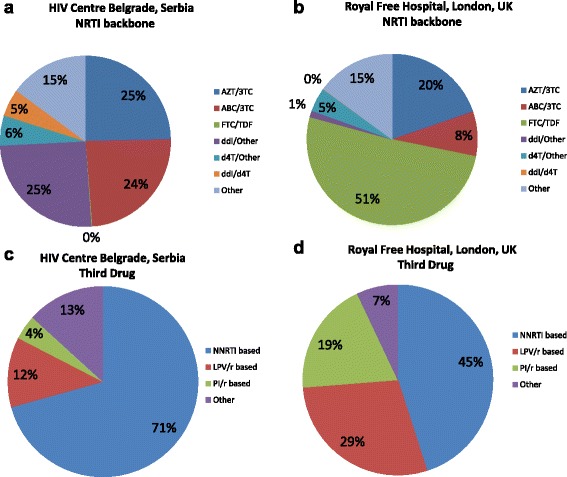
Antiretrovirals included in first-line cART regimen: HIV Centre Belgrade, Serbia and Royal Free Hospital, UK. Antiretrovirals included in first line cART at the HIV Centre Belgrade, Serbia (n = 597; **a** and **c**) and at the Royal Free Hospital, London, UK (n = 1763; **b** and **d**). Choice divided according to nucleoside reverse transcriptase inhibitor (NRTI) backbone prescribed (**a** and **b**) and according to the ‘third’ drug (**c** and **d**) prescribed. AZT – zidovudine, 3TC – lamivudine, ABC – abacavir, FTC – emtricitabine, TDF – tenofovir, ddI – didanosine, d4T – stavudine, NRTI - non-nucleoside reverse transcriptase inhibitor, NNRTI - non-nucleoside reverse transcriptase inhibitor, LPV/r – lopinavir/ritonavir, PI/r – protease inhibitor boosted with ritonavir, cART = combination antiretroviral therapy

There were significant differences in the choice of first-line NRTI backbones at the two centres (p < 0.0001 chi-squared test, Fig. 1a and 1b). At the HCB, the most frequently prescribed NRTI backbone combinations were zidovudine with lamivudine (149; 25 %), didanosine with another NRTI (149; 25 %) and abacavir with lamivudine in (143; 24 %). In contrast, at the RFH the most frequently prescribed NRTI backbones were emtricitabine with tenofovir (899; 51 %), zidovudine and lamivudine (153; 20 %) and abacavir with lamivudine (141; 8 %). At the HCB, NNRTI-based regimens were prescribed for 71 % (423; 55 % [329] efavirenz, 15 % [92] nevirapine and 0.2 % [1] other) versus 45 % at the RFH (793; 39 % [681] efavirenz, 6 % [109] nevirapine and 0.2 % [4] other) (Figs. 1c and 1d; p < 0.0001; chi-squared test).

### Response after 12 months of cART

Twelve months after the start of ART, 21.4 % (95 % CI 17.9 %, 24.9 %) at HCB and 7.4 % (6.4 %, 8.6 %) at RFH had been lost-to-follow-up. There were significant differences in the frequency of CD4 count and pVL monitoring between the two centres. At the HCB, the median (inter-quartile range, IQR) number of CD4+ T-cell counts and HIV-1 RNA pVL measurements in the first year of cART was 2 (1, 2) and 1 (0, 2), respectively; compared to 5 (3, 7; p < 0.0001) and 5 (4, 7; p < 0.0001) at the RFH, respectively.

Three hundred and twenty four (54 %) individuals at HCB had a 12-month CD4 count measurement available, measured at median of 15 months post-cART (75 % [243] were within 12 to 18 months). The median (IQR) was 341 (226, 487) cells/mm^3^, representing a change of +211 (+86, +359) cells/mm^3^ compared to pre-cART values. A substantially higher proportion (1291; 73 %) from RFH had an available CD4+ T-cell counts measurement, taken a median of 14 months after the start of cART (93 % [1206] were within 12 to 18 months). The median value was considerably higher than at HCB, with a value of 437 (296, 580) cells/mm^3^. However, this corresponded to a comparable increase of +212 (+105, +318) cells/mm^3^ compared to pre-cART values.

Of the 597 starting ART at HCB, 287 (48 %) had a VL measurement available after 12 months cART; 113 (19 %) were lost to follow-up prior to this time; 117 (20 %) did not have sufficient follow-up (i.e. started ART less than one year before the study close date), and the remaining 80 (13 %) were under follow-up but without a VL at this time. Of the 1763 starting cART at RFH, 1452 (82 %) had an available VL measurement after 12 months; 123 (7 %) were lost to follow-up, 43 (2 %) did not have sufficient follow-up, and 145 (8 %) were under follow-up but without a VL. Of those with a measurement, 271 (94 %) at the HCB had pVL < 400 copies/mL, compared to 1280 (88 %) at the RFH. If one assumes that those lost-to-follow-up had pVL > 400 copies/ml, then these percentages become 68 % (271/400) at HCB and 81 % (1280/1575) at RFH.

At the end of follow-up (i.e. the last available measurement, the percentage that did not achieve a CD4 count >500 cells/mm^3^ despite achieving a virological response to cART at the HCB was 50 %. A similar discordant response to cART occurred in 36 % patients treated at the RFH.

### First line ARV changes

**Fig. 2:**
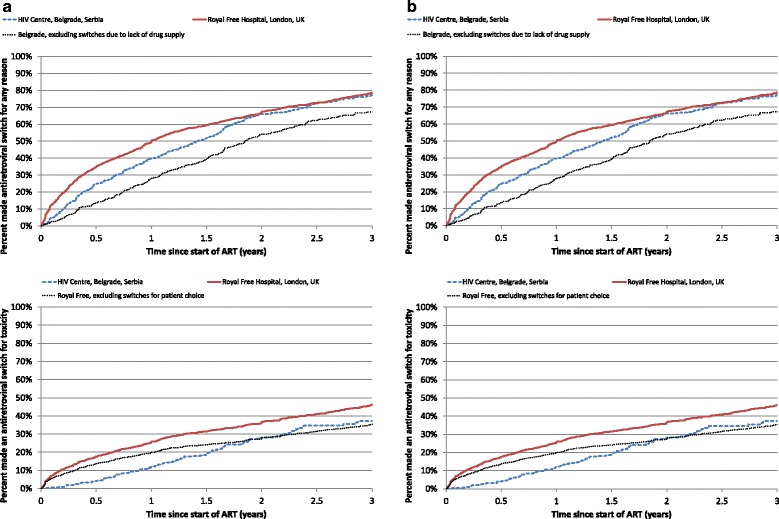
Time to switching an antiretroviral: HIV Centre Belgrade, Serbia and Royal Free Hospital, UK. Kaplan-Meier plot of time from starting cART to the first antiretroviral treatment switch (**a**) for any reason or (**b**) due to toxicity or patient choice. Comparison of HIV Centre Belgrade, Serbia and Royal Free Hospital, London, UK. P-value obtained from log rank test

Over follow-up, 338 (56.6 %) individuals at HCB and 1372 (77.8 %) individuals at RFH made at least one change to their cART regimen (Fig. 2a). At 36 months, the percentage that had made at least one antiretroviral switch to their cART regimen was virtually identical at the two centers (77 %; 95 % CI 72 %, 82 % at the HCB vs. 78 %; 76 %, 81 % at the RFH; p = 0.23). When excluding “lack of drug supply” as a reason for switching at HCB, this centre had considerably lower switching rates compared to RFH (p < 0.0001).

Although overall rates of switching between centers were similar, the reasons given for switching were very different. Considering the 338 first ARV switches at HCB, 109 (32 %) were for lack of drug supply, 58 (17 %) for toxicity [31 peripheral neuropathy, 21 pancreatitis, 6 lactic acidosis], 19 (6 %) for virological failure, 5 (1 %) for dose reduction/ARV simplification and 147 (43 %) for other reasons. In contrast, amongst the 1372 first switches made at RFH, 417 (30 %) were for toxicity [98 CNS disorder, 70 diarrhoea, 39 rash, 33 lipodystophy, 31 anaemia, 30 renal problem, 28 nausea/vomiting, 18 lipid abnormality, 18 deranged liver function tests, 10 abdominal pain, 10 peripheral neuropathy, 32 other known], 170 (12.3 %) for patient choice, 91 (7 %) for virological failure, 326 (24 %) for dose reduction/ARV simplification, and 368 (27 %) for other reasons

As a result, switches made for toxicity and patient choice reasons were considerably more common at the RFH (46 % by 36 months; 95 % CI 43 %, 49 %) than at the HCB (37 %; 31 %, 43 %; *p* <0.0001; Fig. 2b). When excluding patient choice (which was a reason for stopping for RFH only), the percentage making switches were more similar (Fig. 2b).

### Death

**Fig. 3:**
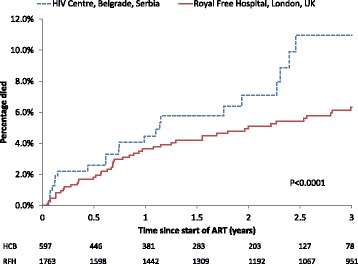
Time from starting cART to death: HIV Centre Belgrade, Serbia and Royal Free Hospital, UK. Kaplan-Meier plot of time from starting cART to death. Comparison of HIV Centre Belgrade, Serbia and Royal Free Hospital, London, UK. P-value obtained from log rank test

By the end of the study period, 31 (5.2 %) deaths had occurred at the HCB and 72 (4.1 %) at the RFH. Mortality at the HCB was significantly higher than at the RFH (p < 0.0001; log rank test). After 12, 24 and 36 months of commencing cART 3 %, 5 % and 8 % of patients had died at the HCB respectively, compared to 2 %, 3 % and 4 % at the RFH (Fig. 3). In order to exclude the effects of late diagnosis on these estimates, this analysis was re-performed restricted to those that started cART in a more timely manner with a CD4 count >200 cells/mm^3^ (n = 249 at HCB and n = 912 at RFH), although this is no longer a representative sample of the complete populations. Differences between the two centres were attenuated although still statistically significant (2.0 % at HCB vs 1.7 % at RFH had died by 36 months; p = 0.006 log-rank test).

## Discussion

Serbia is a middle income, non-European Union country [8], with a relatively low prevalence rate of HIV infection (less than 0.2 %) [9, 10]. Despite this, most HIV-positive individuals are from vulnerable and/or marginalized populations, such as intravenous drug users (IVDUs), commercial sex workers and men who have sex with men [11, 12]. Combination antiretroviral therapy in Serbia is limited and the ability to provide treatment depends on availability of antiretroviral drugs and supply, regardless of current international treatment guidelines. In contrast, the UK, a high-income country and member of the European Union, has a wide range of all registered ARVs together with new experimental drugs. Costs of ARVs are fully covered by National Health Insurance in both cohorts and are free at the point of access [12, 13]. During the study period some cART regimens, abandoned in UK due to increased drug toxicity and decreased viral efficacy, were and are still used in resource-limited settings, such as Serbia [14] as demonstrated in this study.

Most patients from the Serbian cohort had a prior AIDS diagnosis at cART initiation and mean baseline CD4+ T-cell counts below 200 cells*/*mm^3^. In contrast, a lower percentage of individuals from the RFH cohort had an AIDS diagnosis when commencing cART. Unfortunately, information on the exact date of AIDS diagnosis was not available at both centres, but the policy at both was to start cART as soon as practical after diagnosis, in line with treatment guidelines. Our data are consistent with previous studies, strongly suggesting that, in low-middle and middle income countries, cART is usually introduced at an advanced stage of HIV disease, as a direct consequence of low testing rates [14]. In 2006, UNAIDS reported that Serbia had one of the lowest HIV testing rates in Europe, resulting in high percentage of individuals being diagnosed at a late stage of the disease [15]. The Global Fund to fight HIV, tuberculosis and malaria has supported Serbia to undertake National programs to fight HIV/AIDS in order to improve quality of provided health care services and in order to establish conditions for establishing early diagnosis of HIV [16]. Regardless of this, the number of late presenters still remains an issue of concern.

Concerning the first line cART options, significant differences were observed, especially when considering the NRTI component of the regimen. In Serbia, the most frequently prescribed NRTI was zidovudine in combination with lamivudine, followed by didanosine combined with other NRTIs, in comparison to emtricitabine with tenofovir at RFH. Indeed, didanosine was not used at all in study participants from the RFH, due to its exclusion as a recommended first-line drug in national guidelines, and concerns regarding increased toxicity and virological failure. In contrast, didanosine was routinely prescribed in the first regimen at HCB, and some patients remained on the drug throughout follow-up. Use of an NNRTI as the "third" drug of the regimen was more common at the HCB than at the RFH, due to a more limited number of drugs being available in Serbia. Relatively newer drugs, such as tenofovir, emtricitabine, darunavir, tipranavir, etravirine, raltegravir and maraviroc were not available during the study period.

The frequency of CD4+ T-cells count and HIV-1 RNA pVL monitoring varied significantly between those two cohorts. At the HCB, monitoring was below international recommendations for assessment of HIV-positive individuals [17, 18]. The ART-LINC study group, conducted a collaborative analysis in 27 treatment centers in a low and in a middle income countries from Asia, Latin America and sub-Saharian Africa, and found that the frequency of CD4+ T-cell counts testing varied by site, but generally was approximately two tests per year [19]. The EuroSIDA group conducted a sub-study in a resource-limited settings, which revealed more frequent monitoring of CD4+ T-cell counts than of viral loads, which potentially leads to the greater number of virological failures during cART use [20]. These data are consistent with our findings.

As expected, in our study, increases in CD4 count after one year of cART at both centres was substantial and statistically significant. Furthermore, extremely high rates of virological suppression were seen at both centres. These results are compatible with previously published results [17, 18, 21, 22]. Significant improvement in CD4 counts and very high percentages with undetectable in response to cART in the middle income settings, such as Serbia, are still being achieved even though older drugs were used in cART regimens. There are few clinical trials demonstrating the clinical superiority of one regimen over another, instead, licensing bodies rely on CD4+ T-cell counts and HIV-1 RNA plasma viral load as surrogate markers, and that changes in these will ultimately translate into clinical benefits [23, 24]. A number of studies from middle income countries suggest significant issues with immune reconstitution in association with low baseline CD4+ T-cell counts, as long periods of time are needed until counts recover to the normal range of >500 cells/mm^3^ [5–25]. These data are consistent with our study.

After three years of first line cART, there was almost the same frequency of initial ART switch in both centers. However, a lack or interruption of drug supply was the most common reason for treatment change at the HCB, which was never a consideration at the RFH. In a previously reported analysis of the ICONA data [26], the overall risk of discontinuation of first-line cART was 36 % with 21 % due to intolerance/toxicity. In their updated analysis of treatment discontinuation in HIV-1-infected individuals starting their first-line HAART after 2008 they reported the main reason for stopping was simplification, reflecting the recent changes in recommendations aimed to minimize drug toxicity, enhance adherence and improve quality of life [27]. Our study shows that switches for reasons that are not always a “necessity” were much more frequent at the RFH, and reflected tailoring regimens to an individual’s needs including making changes for patient choice. In contrast, at the HCB, such switches were less common due to limited access to alternative regimens. This highlights the fact that individuals from middle-income settings are considerably more likely to need to make switches to their ART regimen and must make these changes more quickly than those from a high income setting, which could potentially be disruptive for the treated patient.

Finally, mortality rates were significantly higher in the HCB than in the RFH cohort, over the first three years after starting cART. Those patients who initiated cART at advanced stage of the disease, which is more frequent in Serbia than in UK, are at increased risk of dying, a finding seen in other similar studies [4, 20, 28]. Our data acts as vital information for health care authorities in the Republic of Serbia as evidence for the need for support of and improvements in testing campaigns. This could lead earlier detection of HIV infection and introduction of cART when individuals are less immune-compromised, both of which have well known clinical benefits. In addition, it highlights the issue of interruptions in the supply chain for antiretrovirals that was present throughout the study period, and which could be improved in the near future. Finally, registration and availability of newer drugs and those still not available at the Republic of Serbia, especially those from the integrase inhibitor class could improve tolerability and reduce the need for drug switching.

## Conclusion

In a middle income country such as Serbia, as a consequence of low HIV-testing rates, antiretroviral treatment is still introduced at an advanced stage of disease. This results in higher mortality rates than in high income countries, such as the UK. Switches of regimen from first line cART are more often due to the lack of drug supplies in such a resource limited setting, while in the high income setting switches are more frequently made in order to tailor regimens to individual patient needs.

## Abbreviations

AIDS: acquired immune deficiency syndrome
ARV: antiretroviral drug
BHIVA: British HIV Association
cART: combination antiretroviral treatment
EACS: European AIDS Clinical Society
HBV: hepatitis B virus
HCB: HIV/AIDS Center of the University Hospital for Infectious and Tropical Diseases in Belgrade, Serbia
HCV: hepatitis C virus
HIV: human immunodeficiency virus
IVDU: intravenous drug user
NNRTI: non-nucleoside reverse transcriptase inhibitor
NRTI: nucleoside reverse transcriptase inhibitor
PI: protease inhibitor
pVL: plasma viral load
RFH: Ian Charleson Centre, Royal Free London Hospital, London, UK
TB: tuberculosis
